# PGRS domain structures: Doomed to sail the mycomembrane

**DOI:** 10.1371/journal.ppat.1010760

**Published:** 2022-09-01

**Authors:** Rita Berisio, Giovanni Delogu

**Affiliations:** 1 Institute of Biostructures and Bioimaging, IBB, CNR, Naples, Italy; 2 Dipartimento di Scienze Biotecnologiche di Base, Cliniche Intensivologiche e Perioperatorie–Sezione di Microbiologia, Università Cattolica del Sacro Cuore, Rome, Italy; 3 Mater Olbia Hospital, Olbia, Italy; Boston Children’s Hospital, UNITED STATES

## Abstract

The impact of artificial intelligence (AI) in understanding biological processes is potentially immense. Structural elucidation of mycobacterial PE_PGRS is sustenance to unveil the role of these enigmatic proteins. We propose a PGRS “sailing” model as a smart tool to diffuse along the mycomembrane, to expose structural motifs for host interactions, and/or to ship functional protein modules at their C-terminus.

## Solving protein structures of pathogenic microbes through artificial intelligence

Like other “revolutions” in science, the release of the artificial intelligence (AI) AlphaFold method to predict protein structure has been initially received with a mix of skepticism and enthusiasm [1]. In just few months, thanks also to the public availability of the training code that allowed to continuously improve the prediction potential while testing the method with a multitude of proteins [2], more than 350,000 protein models from 21 species have been made public, together with smart and effective tools to interrogate their structures [3]. The model accuracy for each protein is expressed as a confidence score for each residue position (predicted local distance difference test (pLDDT)). For example, a very high (pLDDT > 90) confidence score was obtained for 34.8% human protein residues, and for 30.3%, 67.8%, and 72.5% protein residues of *Trypanosoma cruzi*, *Mycobacterium tuberculosis* (*Mtb*) and *Escherichia coli*, respectively. Predicting with high accuracy protein structures of pathogenic microbes as *Mtb* that killed approximately 1.5 million people in 2019, may shed light at molecular level of key pathogenetic mechanisms and pave the way for the development of urgently needed improved therapies and vaccines.

## AlphaFold as a tool to solve the puzzle of the enigmatic PE_PGRS

**Fig 1:**
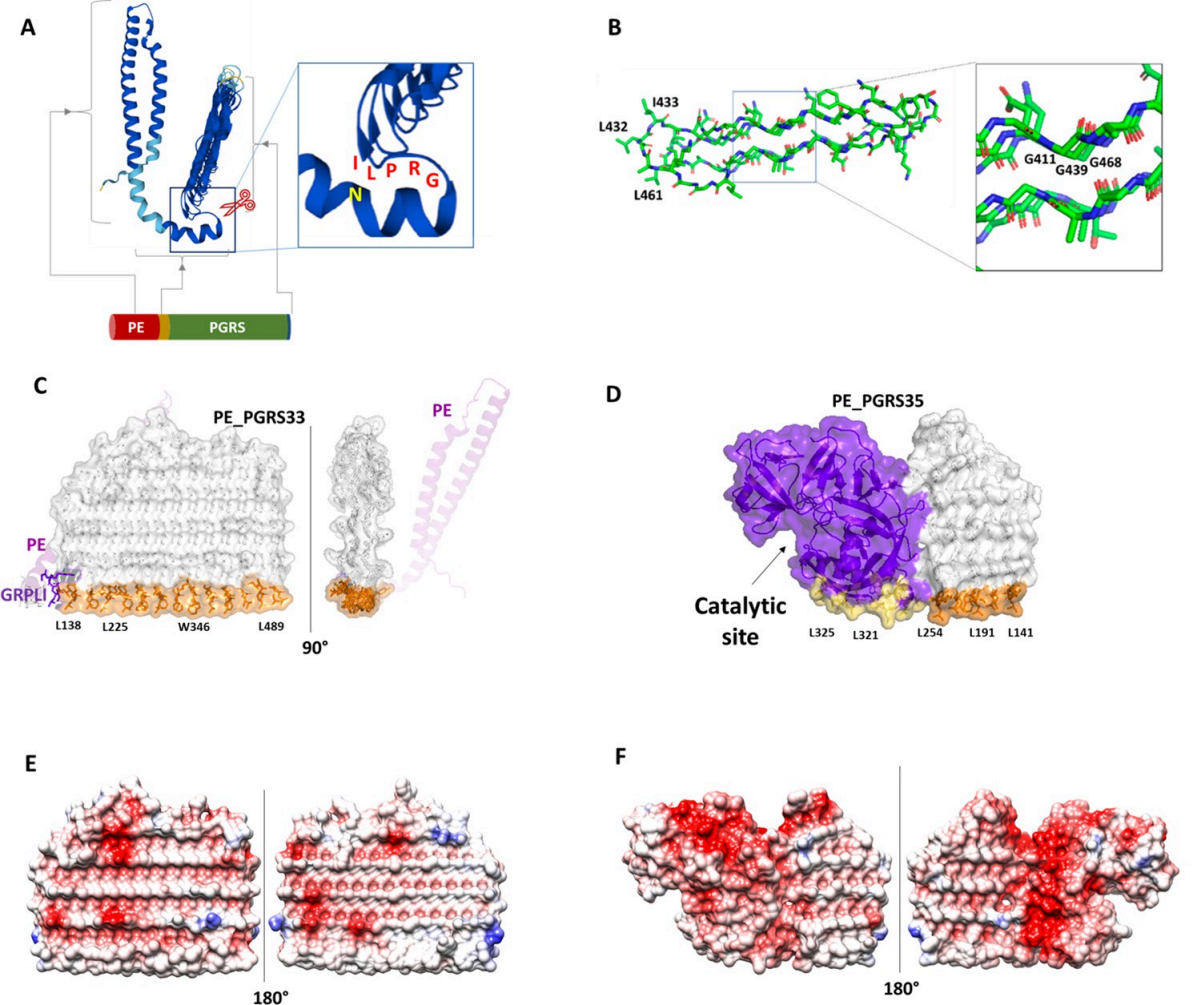
Structural features of PE_PGRS. (A) A schematic view of the organization of a PE_PGRS protein in PE, linker, and PGRS domain. The conserved GRPLI is highlighted. (B) Stick representation of our previously proposed structural model of a PGRS PG_II_ sandwich domain [4,5]; hydrophobic residues on 1 edge and conserved glycine residues pointing inwards the PG_II_ sandwich are labeled. (C, D) Structural representation of PE_PGRS33 and PE_PGRS35 structures, respectively. PE_PGRS33 PE domain is shown in magenta cartoon, the PGRSs domains in white stick/surface, the PE_PGRS35 catalytic domain in prune cartoon/surface. Hydrophobic residues lining the straight edges of the PGRS33 and PGRS35 domains are colored orange, those of PE_PGRS35 catalytic domain in yellow. (E, F) Two 180° views of electrostatic potential surfaces of PGRS33 and PGRS35, respectively.

Approximately 10% of the *Mtb* genome is occupied by the PE and PPE genes, coding for proteins that are unique for *Mtb* and few other pathogenic mycobacteria [4,5]. Among these, the PE_PGRS and PPE_MPTR subfamilies are peculiar for the apparently redundant and extensive amino acid sequences downstream the PE and PPE N-terminal domains, respectively [5]. PE_PGRSs are a large family of *Mtb* proteins implicated in tuberculosis (TB) pathogenesis that show a modular structure [6,7] (Fig 1A): the N-terminal PE domain, highly homologous to the domain found in tens of others PE proteins and whose structure has been already solved by crystallography [8]; a C-terminal domain that is unique for each protein; the polymorphic glycine-rich domain (PGRS) that varies in length from few tens to more than 1,000 amino acids and whose structure has puzzled scientist for more than 20 years. The difficulties in expressing and purifying in native conditions PE_PGRS proteins has so far prevented experimental structural studies. We have recently proposed a structural model for PGRS domains, in which glycine-rich triplets fold into left-handed helices to form poly-glycine II (PG_II_) sandwiches (Fig 1B) [6]. The model explained the puzzling high abundance of glycine residues in these domains, as glycine is always pointing inwards and is the sole residue to be sterically allowed (Fig 1B). In small PG_II_ sandwiches, 5 or 6 antiparallel PG_II_ helices are stacked in 2 antiparallel groups, with 3 to 4 triplets spanning the PG_II_ domain length (about 35 to 50 Å). The proposed model was also consistent with a plastic structure that can tolerate large indels while maintaining correct localization on the mycomembrane, providing a structural and functional framework for the polymorphic PGRS domain [9]. Recently, AlphaFold predicted the structure of many *Mtb* proteins, including PE_PGRSs, offering a unique opportunity to discuss how the findings obtained on these structurally complex and enigmatic proteins can be illuminating to better understand their role in TB pathogenesis and may serve as a model to solve key questions in biology [10].

## The predicted structure supports the model claiming cleavage of the PE domain by a specific protease

AlphaFold recently released the structures of several PE_PGRS proteins, most of which with a high confidence score. The PE domain predicted structure is consistent with that proposed for PE/PPE couples and for many other PE proteins [8,11]. The polymorphic linker region found immediately downstream the PE domain is generally predicted as an α-helix extending up to the highly conserved GRPLI motif (Fig 1A). Similarly to what observed for other PE proteins, the linker domain seems properly positioned for recognition by EccC_5_, the cytoplasmatic module of the type VII secretion system ESX-5, with the polymorphisms between the different PE_PGRS possibly providing specificity for secretion [11,12]. Recent evidence indicates that the PE domain of PE_PGRSs is cleaved off by a protease that in *M*. *marinum* has been identified in PecA, the homologous of PE_PGRS35 in *Mtb* [13]. The protease domain of PE_PGRS35 localizes in the unique C-terminal domain that is positioned to recognize and cleave off the tubular-like PE domain (Fig 1A). Experimental data indicate that cleavage of PE_PGRS proteins remove approximately 11 kDa fragment, corresponding to approximately 110 N-terminal amino acids [13,14], though the exact cleavage site has not yet been identified. These observations, together with the predicted structure of the PE_PGRSs (see below), suggest that cleavage of the PE domain may occur upstream the PGRS domain. In keeping with these observations, PE_PGRSs proteins localize on the mycomembrane outer leaflet with their PGRS and the unique C-terminal domains available on the mycobacterial surface [6,9,13].

## AlphaFold prediction supports the PG_II_ sandwich model of PGRS

**Fig 2:**
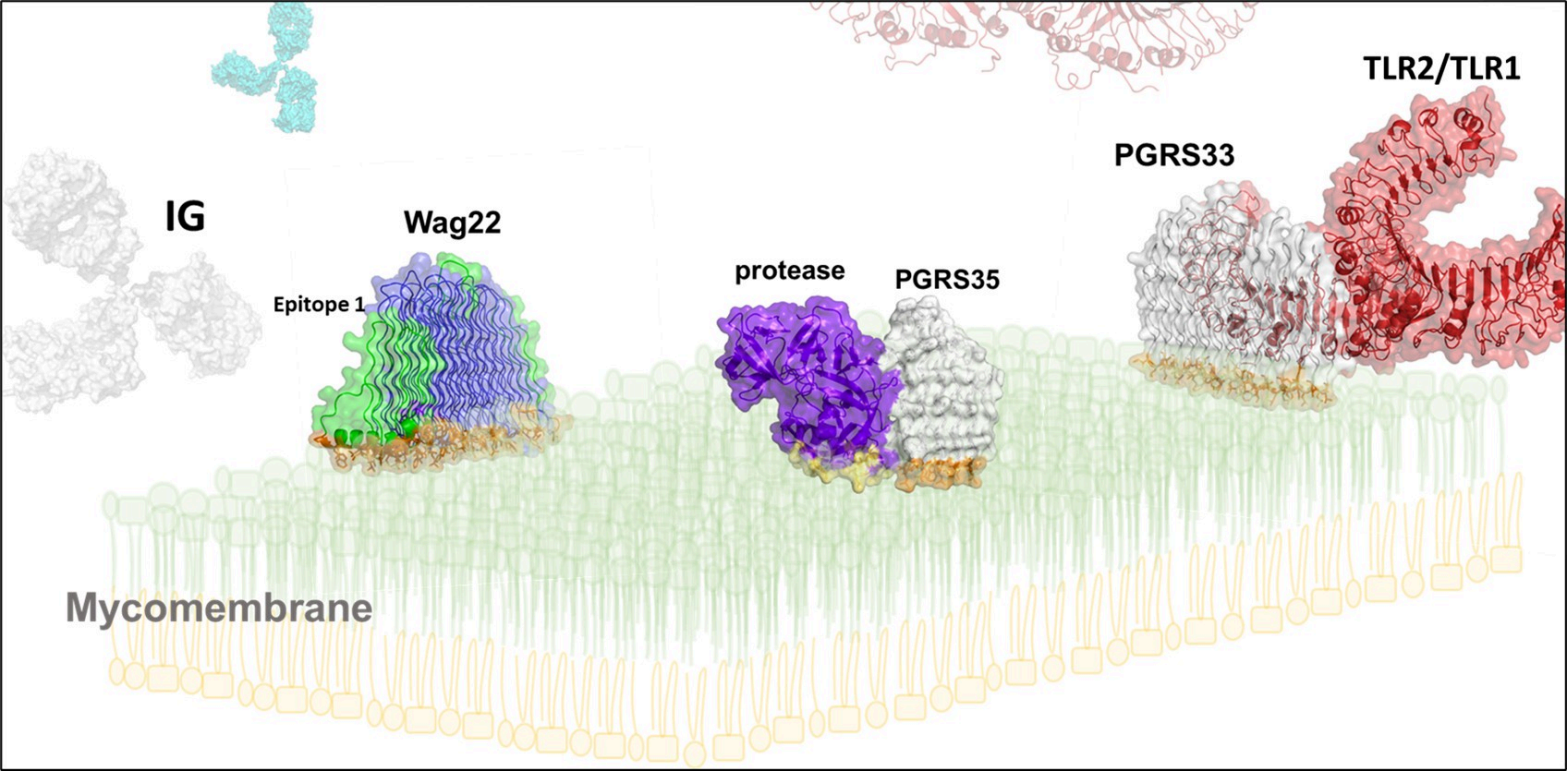
A speculative drawing of PE_PGRS33 (white), −35 (white-prune), and Wag22 (blue-green) proteins sailing the mycomembrane. TLR2 (pdb code 6nig) was docked on PGRS33 structure by combining surface complementarity, using the software PatchDock, with refinement of electrostatic interactions and desolvation energy, using FireDock. Structure-based B cell antigen prediction was performed using ElliPro.

AlphaFold predicts the PGRS domain as tightly packed sandwiches that are consistent with the PG_II_ sandwich model we recently proposed [6,9]. There is a large variability in the number of PG_II_ helices that compose PGRS sandwiches, from the small sandwiches of PE_PGRS17, −18, −11, and −35 (7, 10, 11, 11 helices) to the medium sized as in PE_PGRS33 and −47 (27 and 29, respectively) to the large ones as in Wag22 (54 helices). However, they all share the same peculiar structural features: (i) they are all flat and sail shaped, with 1 straight edge made of short and regular loops rich in hydrophobic and aromatic residues as Phe and Trp and less frequently Tyr (Fig 1C and 1D); (ii) on the opposite side, they exhibit an irregular edge exposing loops of variable amino acid composition; and (iii) their 2 lateral sides are characterized by negative electrostatic potential surfaces (Fig 1E and 1F). Interestingly, some PE_PGRS proteins as PE_PGRS3 or Wag22 are characterized by the presence of 2 GRPLI motifs and present 2 parallel sails (www.uniprot.org/uniprotkb/P9WIG5/entry). Altogether, structural features shared by all PGRSs strongly suggest that the straight hydrophobic edge, in the absence of trans-membrane helices, is a smart tool to embed these domains in the mycobacterial outer leaflet, thus pointing the opposite irregular edge outward the mycomembrane (Fig 2). Indeed, structures of PGRS domains share the typical “protruding hydrophobes” that were proposed as a discriminating criterium to identify protein surfaces involved in membrane binding and therefore fingerprints of peripheral proteins [15]. Importantly, the negative electrostatic potential surface of the 2 sides of the PGRS sails may constrain them to be orthogonally aligned to the mycomembrane, due to their unfavorable electrostatic interactions with the negatively charged mycomembrane. As a result, mature PGRSs associate to outer leaflet through nonspecific hydrophobic interactions, which make PGRS domains free sailors on the “fluid” mycomembrane [16], where they can expose moieties conferring specific functions to each PE_PGRS.

## Experimental evidence from single PE_PGRS support the “sailing” model

PE_PGRS33, one of the most investigated proteins, is known to mediate entry into macrophages through TLR2 interactions [17]. TLR2 can dimerize to form heterodimers with TLR1 or TLR6 depending on the interaction with acylated lipoproteins, though several studies indicate that the TLR2/TLR1 heterodimer is more often involved in the detection of TLR2-specific mycobacterial ligands [18], classically represented by triacylated lipopeptides that bind to the hydrophobic pocket in TLR2 [19]. In the docking model we propose, the PGRS domain of PE_PGRS33 interacts with the externally exposed amino acids located in the proximity of the hydrophobic ligand-binding pocket of TLR2 (Fig 2), without hindering the binding of the classical TLR2 ligands to the hydrophobic pocket. Given the experimentally demonstrated ability of PGRS33 to bind TLR2 and activate the signaling pathway via MyD88, it remains to be determined whether the interaction between the PGRS33 is sufficient to promote heterodimerization of TLR2 with TLR1 (or TLR6) and activate the signaling pathway in absence of the ligand or if PGRS33/TLR2 interaction promotes or facilitates binding of the ligand to the hydrophobic pocket. While we consider more likely the latter hypothesis, it would be highly speculative to propose a model in absence of sufficient data. Yet, regardless of the precise mechanism, the exposure of the PGRS sail on the mycomembrane well agrees with a role of PE_PGRS33 in engaging TLR2 through interactions with its PGRS33 sail (Fig 2) [17,20]. In line with this model, the PGRS domain of PE_PGRS5 can target the endoplasmic reticulum and promote a TLR4-dependent cell death [21,22], yet the low confidence score obtained by AlphaFold in the prediction of the PE_PGRS5 structure prevents any speculation on the region involved in the interaction with TLR4. Similarly, the PGRS domain of PE_PGRS31 interacts with S100A9 factor in macrophages to promote mycobacterial survival [23]. Similarly, our sailing model ensures an effective exposure of epitope regions in the PG_II_ sandwich structure of the highly antigenic Wag22 [24] and similar considerations can be extended to other PE_PGRS proteins that have been proposed to serve as immunological decoys [25]. Indeed, the fact that PGRS domains are endowed with a well-structured fold make them even more suitable to serve as decoys, as they may expose structural motifs, with specific conformations, that act in the camouflage of effector molecules responsible for immune evasion [25].

Another interesting example is PE_PGRS35, which contains a C-terminal aspartic protease domain able to hydrolytically deprive other PE_PGRS proteins of their PE domains (Fig 1D). In this specific case, our suggested feature of PGRS35 to root in the mycomembrane through its hydrophobic edge (Fig 1D) may be functional to the proper orientation of the protease domain for catalysis and to allow it to easily float on the mycomembrane to meet its substrates (Fig 2). This may also be the case for the few other PE_PGRS proteins with a unique C-terminal domain endowed with specific functions (as outlined in S1 Table). These PE_PGRSs may be considered moonlight proteins, with the PGRS domain providing proper cellular localization to the whole protein and playing a specific role thanks to the moieties exposed outward in the PGRS domain and the unique C-terminal domain exerting the enzymatic activity or its peculiar function.

In conclusion, new structural evidence provides unprecedented clues on the role of PGRS domains in mycobacterial life. As in the old legend of the Flying Dutchman, PGRS domains are doomed to sail the mycomembrane without making port; PGRS domains are molecular sailors that allow mycobacteria to sense or interact with host molecules and/or to ferry mycobacterial enzymes or other functional protein domains across the mycomembrane.

## Supporting information

S1 Table Predicted functions of PE_PGRS C-terminal domains based on structure alignment. Z-score and root mean square deviations (RMSD) from most similar structures were computed using DALI.
(DOCX)
