# Supplementary material for: PGRS domain structures: Doomed to sail the mycomembrane
Source: PLoS Pathog. 2022 Sep 1;18(9):e1010760. doi: 10.1371/journal.ppat.1010760 (PMC9436101; doi:10.1371/journal.ppat.1010760)
Supplement: S1 Table — Z-score and root mean square deviations (RMSD) from most similar structures were computed using DALI. (DOCX) [file ppat.1010760.s001.docx]

|  | **Total N. of residues** | **Residues of the C-terminal domain** | **DALI**  **Z score** | **RMSD (Å)** | **Aligned residues** | **Sequence identity (%)** | **PDB code of reference structure** | **Predicted function** | **Reference** |
| --- | --- | --- | --- | --- | --- | --- | --- | --- | --- |
| PE_PGRS3 | 957 | 875-957 | - | - | - | - | - | **Phospholipid binding*** | [2] |
| PE_PGRS11 | 584 | 279-584 | 22.1 | 2.6 | 194 | 21 | 6s2r | **Phosphatase** | <https://pdbj.org/emnavi/quick.php?id=pdb-6s2r> |
| PE_PGRS16 | 1081 | 653-1081 | 37.2 | 1.8 | 267 | 97 | 4ehc | **Aspartic proteinase** | [3] |
| PE_PGRS17 | 327 | 209-327 | 13.4 | 1.4 | 119 | 45 | 1l0q | **S-layer protein** | [4] |
| PE_PGRS18 | 457 | 284-457 | 16.2 | 1.7 | 169 | 41 | 1l0q | **S-layer protein** | [4] |
| PE_PGRS35 | 558 | 266-558 | 29.3 | 1.9 | 253 | 47 | 4ehc | **Aspartic proteinase** | [3] |

**S1 Table.** Predicted functions of PE_PGRS C-terminal domains, based on structure alignment. Z-score and root mean square deviations (RMSD) from most similar structures were computed using DALI ([1])

**In this case, the C-terminal region is an α-helix and not a tertiary structure. Therefore, function prediction was based on the observation of its arginine-rich nature. This suggested us binding to phospholipids, as we experimentally assessed (De Maio et al. 2021)[2].*

**References**

1. Holm L. Using Dali for Protein Structure Comparison. Methods Mol Biol. 2020;2112:29-42. doi: 10.1007/978-1-0716-0270-6_3. PubMed PMID: 32006276.
2. De Maio F, Salustri A, Battah B, Palucci I, Marchionni F, Bellesi S, et al. PE_PGRS3 ensures provision of the vital phospholipids cardiolipin and phosphatidylinositols by promoting the interaction between M. tuberculosis and host cells. Virulence. 2021;12(1):868-84. doi: 10.1080/21505594.2021.1897247. PubMed PMID: 33757409; PubMed Central PMCID: PMCPMC8007152.
3. Barathy DV, Suguna K. Crystal structure of a putative aspartic proteinase domain of the Mycobacterium tuberculosis cell surface antigen PE_PGRS16. FEBS Open Bio. 2013;3:256-62. doi: 10.1016/j.fob.2013.05.004 [doi];FOB97 [pii].
4. Jing H, Takagi J, Liu JH, Lindgren S, Zhang RG, Joachimiak A, et al. Archaeal surface layer proteins contain beta propeller, PKD, and beta helix domains and are related to metazoan cell surface proteins. Structure. 2002;10(10):1453-64. doi: 10.1016/s0969-2126(02)00840-7. PubMed PMID: 12377130.
